# Multimodality Management of EBV-Associated Nasopharyngeal Carcinoma

**DOI:** 10.3390/cancers13236078

**Published:** 2021-12-02

**Authors:** Justin Yu, Tiffany T. Pham, Narine Wandrey, Mackenzie Daly, Sana D. Karam

**Affiliations:** 1 Department of Otolaryngology, Head and Neck Surgery, University of Colorado, Anschutz Medical Campus, Aurora, CO 80045, USA; justin.yu@cuanschutz.edu (J.Y.); tiffany.pham@cuanschutz.edu (T.T.P.); 2 Department of Radiation Oncology, University of Colorado, Anschutz Medical Campus, Aurora, CO 80045, USA; narine.wandreybhardwaj@cuanschutz.edu (N.W.); mackenzie.daly@cuanschutz.edu (M.D.)

**Keywords:** HNSCC, nasopharynx, radiation therapy, immunotherapy, tumor microenvironment, cancer immunology

## Abstract

**Simple Summary:**

Nasopharyngeal carcinoma (NPC) is a cancer that arises from the mucosal epithelium of the nasopharynx. NPC is usually detected at a locoregionally advanced stage, resulting in the need for multimodality therapy. This review highlights the existing clinical trials investigating the optimal chemoradiation regimens. A specific focus was made to describe the latest clinical trials regarding the use of induction chemotherapy, which has now emerged as a mainstay of treatment. NPC also has a unique biologic signature that is associated with the Epstein-Barr Virus (EBV). Immunotherapy drug development has centered around exploiting the immune-cell rich tumor microenvironment by instigating the use of immune checkpoint blockage therapies. The close association of NPC with EBV has led to the identification of specific biomarkers that allow for real-time monitoring of disease response and prognostication, resulting in a potential new era of precision and personalized medicine.

**Abstract:**

Nasopharyngeal carcinoma (NPC) is a rare cancer of the nasopharyngeal mucosa with a specific geographic predisposition. NPC is often associated with Epstein–Barr Virus (EBV) infection and as a result contains many characteristic biomarkers. Treatment of locally-contained NPC is generally achieved through use of radiotherapy (RT), as part of a multimodality treatment regimen. Induction chemotherapy followed by concurrent RT and platinum-based chemotherapy regimen has emerged as the definitive treatment of choice for locoregionally-advanced NPC. Recently, immunotherapy is finding a role in the treatment of recurrent or metastatic NPC. Immune checkpoint blockade therapies targeted against the programmed death-1 (PD-1) receptor have demonstrated efficacy in early phase clinical trials, with ongoing phase III trials in effect. Biomarkers for treatment efficacy remain an ongoing area of investigation, with important prognostic implications on the horizon.

## 1. Introduction

Nasopharyngeal carcinoma (NPC) is a rare malignancy that arises from the epithelial layer of the nasopharyngeal mucosa. In 2018, there were estimated to be approximately 129,000 new cases of NPC, accounting for 0.7% of all newly diagnosed cancers worldwide [1]. NPC has a skewed geographic and ethnic predilection, with higher prevalence in southeast Asia, southern China, and north Africa [1]. In addition to environmental, lifestyle, and genetic factors, the Epstein–Barr virus (EBV) is a known contributor to the pathogenesis of NPC [2]. NPC is characterized by extensive local invasion and hematogenous spread, and treatment therefore must be tailored to the disease burden.

Treatment of NPC is continuing to evolve with greater understanding of the disease process. NPC has been found to be highly sensitive to radiotherapy (RT), which now often serves as the mainstay modality for early stage, non-metastatic disease. Intensity-modulated radiotherapy (IMRT), which can precisely deliver high doses of radiation sparing adjacent structures, has replaced conventional 2D and 3D RT as the treatment of choice [3], due to improved locoregional disease control as well as improved overall survival (OS) [4,5]. 

However, it is estimated that up to 70% of patients are diagnosed with locoregionally-advanced disease at initial presentation [6]. As a result, RT alone is often not sufficient in controlling their advanced disease. Studies have shown improved OS with combined regimens of chemotherapy and RT. The landmark Intergroup-0099 study performed in 1998 showed improved OS with treatment of concomitant chemoradiotherapy (CRT) compared to RT alone [7]. Further meta-analyses (MAC-NPC-1 and MAC-NPC-2) have demonstrated significant survival benefits using concurrent CRT delivered with and without adjuvant chemotherapy [8,9]. Definitive RT followed by adjuvant chemotherapy was not found to improve OS [9]. 

Induction chemotherapy (IC) given prior to concurrent CRT is an ongoing area of investigation. Compared to adjuvant chemotherapy, IC is better tolerated and thought to treat early micro-metastases. Recent clinical trials have shown promising results for induction chemotherapy followed by concomitant CRT. This review will discuss the role, principles, and future directions of induction chemotherapy in the treatment of NPC. Moreover, new breakthroughs in immunotherapy suggest that immunomodulation may play a large role in the management of refractory or metastatic disease. Ongoing trials for concurrent immunotherapy and the use of biomarkers to predict treatment response will also be discussed. 

Our review highlights the latest developments in the treatment of NPC with a focus on novel clinical trials regarding induction, immunotherapy, and the role of biomarkers. Our review was conducted via the following search strategy on PUBMED: “nasopharyngeal carcinoma” and “induction therapy” or “immunotherapy” or “tumor microenvironment” or “biomarkers”.

## 2. Induction Therapy

The general goal of IC is to improve the sensitivity of tumors to subsequent treatments such as surgery and/or CRT by eradicating micro-metastatic disease and decreasing tumor burden [10]. Investigation of IC use in head and neck cancers has been ongoing for decades. Recent improvements in chemotherapy regimens, such as implementation of the docetaxel (Taxotere), cisplatin (Platinol), and fluorouracil (TPF) triplet, have also shown promising results in increasing OS and progression-free survival (PFS) in select populations of head and neck squamous cell carcinomas (HNSCC) [10,11]. In addition, newer induction regimens have reduced toxicity profiles compared to those of earlier regimens, and have been correlated with improved patient tolerance and compliance [11,12]. 

The initial foray of IC for the treatment of NPC was met with equivocal results. Two phase II clinical trials were conducted and completed in the mid-2000s. Hui et al. used docetaxel-cisplatin as the neoadjuvant regimen and found a statistically significant improvement in 3-year OS, but not in PFS in 65 randomized participants [13]. Fountzilas et al. randomized participants to neoadjuvant cisplatin–epirubicin–paclitaxel followed by CRT versus CRT alone. This study did not find statistically significant improvement in either 3-year OS or PFS [14]. One of the first phase III clinical trials examining IC in treatment of NPC, which was published in 2015 by Tan et al. [15], investigated the use of induction gemcitabine–carboplatin–paclitaxel. This study of 180 randomized participants similarly did not detect a difference in 3-year OS or PFS between the investigational and control arm. 

Critiques of the initial IC studies in NPC were focused on limited sample size and inconsistencies between the induction regimens. Studies investigating the use of the TPF regimen (docetaxel–cisplatin–fluorouracil) in IC for locally-advanced NPC have been encouraging. Recently in 2016, Sun et al. published a large, multi-center phase III study from 10 institutions in China that enrolled a total of 480 patients who were randomized to receive IC with TPF prior to CRT or standard concurrent CRT in 1:1 ratio [16]. The results showed an improved OS (hazard ratio (HR) 0.68, 95% CI 0.48–0.97; *p* = 0.034) and PFS (HR 0.65, 95% CI 0.43–0.98; *p* = 0.019) in the treatment group versus the control. Subsequently, a small phase III trial in Tunisia/France, studying 83 patients, used the same induction regimen and found a similar improvement in 3-year PFS (HR 0.44, 95% CI 0.20–0.97; *p* = 0.042) but a borderline significant 3-year OS (HR 0.40, 95% CI 0.15–1.04; *p* = 0.059) [17].

The most recent phase III clinical trial, published by Zhang et al. in May 2019, suggested improved survival benefits when using gemcitabine IC therapy. A total of 480 patients with advanced stage (III or IV) NPC were randomized to receive either gemcitabine induction therapy plus concurrent CRT or concurrent CRT alone. The gemcitabine group was found to have improved 3-year OS of 94.6% versus 90.3% (HR 0.43, 95% CI 0.24–0.77) and improved 3-year recurrence-free survival of 85.3% versus 76.5% (HR 0.51, 95% CI 0.34–0.77) [18]. This study stands in contrast to the negative results reported by Tan et al., which were described earlier. It is hypothesized that the negative result of the Tan study was possibly due to a lower disease burden (fewer patients with N2 and N3 disease), resulting in cancer more amenable to concurrent CRT treatment only, and the use of low-dose concurrent carboplatin which may have compromised the synergy with gemcitabine [15]. 

There have been reports of increased treatment-related toxicity from induction therapy followed by CRT compared to that of CRT alone. A recent meta-analysis of randomized clinical trials demonstrated improved survival benefit with IC, but high incidence of toxic reactions and patient discomfort [19]. In particular, IC may confer a higher risk of neutropenia, thrombocytopenia, nephrotoxicity, and nausea/vomiting [16,18,20]. There are ongoing studies evaluating optimization of IC, including dose reduction and/or the number of treatment cycles [21]. Currently, there is no definitive consensus regarding optimal intensity of IC regimens [22]. 

**Figure 1:**
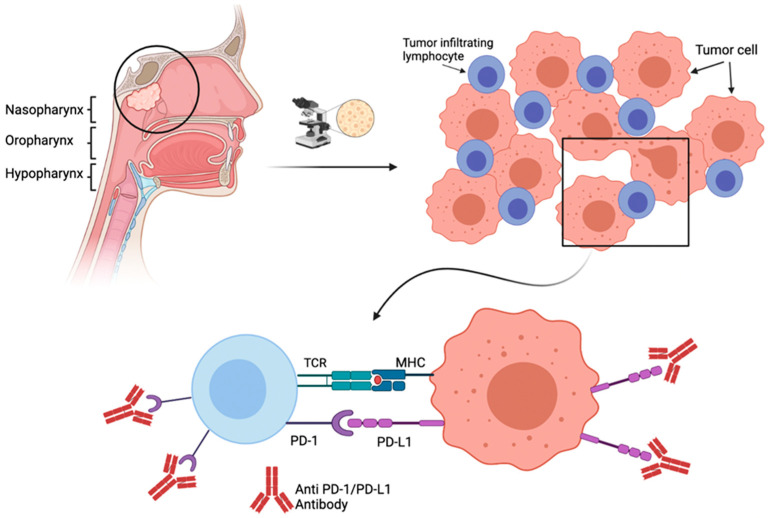
Tumor microenvironment of immune-rich nasopharyngeal carcinoma (NPC). NPC is often associated with heavy lymphocyte infiltration in a histologic setting. Tumor cells express high levels of PD-L1 as a way to evade the adaptive immune system, which have since served as targets for immunomodulation therapies.

The tumor microenvironment (TME) has recently come to the forefront of attention. Multiple studies have sought to better characterize the TME of NPC, in hopes of finding biomarkers which could have both prognostic and predictive value. EBV is a known instigator of NPC and associated with a heavy lymphocytic infiltrate on a microscopic level in the host’s normal tissues [23]. Recent work has detected differences in TME between various types of NPC. Certain subsets of NPC have an immune-rich TME, comprising of high concentrations of host immune cells (T-cells, B-cells, macrophages) as well as increased expression of checkpoint inhibitor proteins such as programmed death ligand 1 (PD-L1) and transforming growth factor (TGF)-beta [24,25,26] (Figure 1). It is hypothesized that these immune-rich NPCs may be associated with more favorable outcomes and are more readily targeted by new therapies such as immunotherapy. The reader is referred to the excellent review that was recently published on the topic and provides a comprehensive review of the complexity of the TME within NPC [24].

## 3. Immunotherapy

Treatment of metastatic NPC has evolved over time with the development of new breakthrough therapies. Platinum-based chemotherapy regimens, as discussed previously, remain the first-line treatment for metastatic NPC. Zhang et al. demonstrated that gemcitabine plus cisplatin is an effective first-line treatment for recurrent or metastatic disease [18]. However, recent developments in the field of immunotherapy have contributed to promising new therapeutic approaches for the management of metastatic NPC that may be refractory to CRT. The principles of immunotherapy in the treatment of NPC center around strategies of EBV-directed immunization, adoptive T-cell therapy, and immune checkpoint blockade. 

EBV is a known instigator in the transformation of nasopharyngeal mucosa into cancerous growth. Previous vaccine design strategies have centered around mimicking EBV- specific proteins that are expressed in NPC, including EBNA1, latent membrane protein 1 (LMP1), and LMP2 [27]. Phase 1 trials in the US and UK demonstrated development of EBNA1-specific and LMP2-specific T lymphocytes which followed a dose response curve [28,29]. A phase 2 trial is underway and is completing data collection (NCT01094405).

More recently, immune checkpoint blockade therapies have achieved large breakthroughs in the treatment of NPC. Programmed death ligand 1 (PD-L1) expression is a characteristic finding of EBV-associated malignancies [30] (Figure 1). Studies theorize that the high expression of PD-L1 on these cancer cells allows for evasion of the adaptive immune system through downregulation of T-cell response [31]. The use of monoclonal antibodies to target PD-1 receptors has shown favorable clinical outcomes for metastatic or refractory NPC in numerous studies [32,33,34,35]. 

The first phase 1 study was conducted by Hsu et al. in 2017 for a cohort of 27 participants with either metastatic or unresectable disease, which progressed despite treatment with standard-of-care therapy [32]. Pembrolizumab, a monoclonal antibody against PD-1, produced a 26% objective response rate in this study, as well as a 63% 1-year OS, and 33% 1-year PFS benefits [32]. Ma et al. conducted a phase 2 trial which examined nivolumab in 44 participants and demonstrated similar results, with an objective response rate of 21%, and 1-year OS and PFS of 59% and 19%, respectively [33]. 

Fang et al. reported on two trials where camrelizumab, another PD-1 inhibitor, was used as monotherapy and then as combination therapy with gemcitabine plus cisplatin [34]. As monotherapy, camrelizumab was found to have an objective response rate of 34% with 1-year PFS of 27%, which is in line with prior studies. However, when used in conjunction with gemcitabine/cisplatin, the objective response rate increased to 91% with an increased 1-year PFS of 61% [34]. This sparked subsequent interest in studying the role of adding immunotherapy to existing CRT regimens. 

Numerous phase 3 clinical trials have been undertaken to investigate the efficacy of adding immunotherapy to current CRT regimens. One of these trials (NCT03581786) has recently finished data collection and published preliminary data. In a sample of 289 patients, 146 were randomized to toripalimab (PD-1 inhibitor) and gemcitabine/cisplatin, whereas the remaining 143 patients were treated with gemcitabine/cisplatin alone. Preliminary analysis demonstrated promising results. PFS was found to be improved in the toripalimb arm (11.7 months vs 8.0 months, 95% CI: [0.36–0.74], *p* = 0.0003), and the 1-year PFS rate was 49% compared to 28% in the control arm [35]. OS per the preliminary report is still being analyzed, but these early results appear promising for the addition of immunotherapy to existing CRT regimens. A synopsis of these studies and their results can be found in Table 1.

## 4. Biomarkers

Biomarkers associated with NPC are unique due to its association with EBV. Existing studies have evaluated the role of DNA methylation, microRNA (miRNA) expression, and cell-free EBV DNA on predicting prognostic outcomes.

Emerging evidence has pointed to the importance of epigenetics in oncogenesis. DNA methylation is a common epigenetic change, and methylation changes can lead to inactivation of gene expression. There has been evidence that gene methylation in epithelial cells can lead to the carcinogenesis of NPC [36,37]. One bio-informatics study examined a large cross section of NPC gene expression to identify certain gene hubs with aberrant methylation patterns that were linked to differences in overall survival [38]. Moreover, Jiang et al. identified a six-hypermethylation gene panel that had an unfavorable response to concurrent chemotherapy and was thus associated with poor survival outcomes in NPC [39]. 

In addition, miRNA dysregulation has been identified as a significant factor in the pathogenesis of cancer formation. Many human miRNA have been found to play crucial roles in regulating the cell cycle, cell proliferation and apoptosis, and tumor angiogenesis [40,41,42]. Studies have shown that dysregulation of specific miRNA sequences can be predictive of radiosensitivity [43] and chemosensitivity [44]. A five-miRNA signature was identified by Liu et al. which was found to be a prognostic indicator of disease-free survival, independent of TNM staging [45]. A separate four-miRNA signature was developed by Zhang et al. and found to be predictive of patient outcomes [46]. These outcomes taken together indicate that biomarkers will likely play a large role in the future of precision medicine going forward.

Real time PCR tests have been devised to quantify the amount of cell-free EBV DNA in blood. Several studies have shown that high concentrations of EBV DNA are associated with poorer prognosis [47,48]. Moreover, post-radiotherapy EBV DNA levels may also correlate with recurrence rates and have been studied as a marker for disease surveillance [49,50]. It is important to note that due to methodologic differences in molecular EBV DNA quantification assays, direct comparisons between clinical studies can often be challenging.

Huang et al. demonstrated that low plasma EBV DNA load after IC was a predictor of improved response to therapy [51]. This raised the question of whether cell-free DNA concentrations could be used to personalize medical treatments in real time. Chan et al. were the first to conduct a randomized clinical trial on using EBV DNA to guide decisions on administering post-RT adjuvant chemotherapy [52]. Though this study was unable to detect a difference in outcomes for biomarker-driven adjuvant chemotherapy versus observation alone, it argued for a novel approach using in-treatment EBV DNA response for risk stratification and personalized treatment. We are still awaiting the results of a larger clinical trial (NCT02135042) that is investigating de-intensification of systemic therapy based on post-CRT EBV DNA levels. As new data begin to emerge, the implementation of EBV DNA and other biomarker approaches may entail a new era of personalized medical therapy for NPC.

## 5. Conclusions

The treatment of EBV-associated NPC continues to evolve over time. While RT remains the convention for locally-contained disease, IC followed by concurrent CRT has emerged as the standard of care. Ongoing research aims to optimize IC regimens, with the hope of mitigating treatment-related toxicity. Immunotherapy has also begun to play a larger role in the management of recurrent and metastatic NPC. Existing studies have shown positive effects of immunotherapy, most notably immune checkpoint regulators such as PD-1 inhibitors, on salvage cases of metastatic NPC. Ongoing clinical trials are attempting to characterize the role of concurrent immunotherapy with chemotherapy for recurrent or metastatic disease. As new tumor biomarkers begin to emerge, future research is needed to better predict immunotherapy response to specific tumor markers.

## Figures and Tables

**Table 1:** Compilation of completed and ongoing phase 1–3 clinical trials investigating efficacy of immunotherapy in advanced NPC.

Author	Phase	Eligibility	Immunotherapy Regimen	Control Regimen	Sample Size	Objective Response Rate	OS (1 Year) (%)	PFS (1 Year) (%)
Phase 1/2 completed studies								
Hsu et al.	1	Unresectable or metastatic disease, failure on prior standard therapy, and PD-L1 expression in 1% or more of tumor cells or tumor-infiltrating lymphocytes	Pembrolizumab 10 mg/kg every 2 weeks up to 2 years or until disease progression or unacceptable toxicity	N/a	27	26%	63	33
Ma et al.	2	Recurrent or metastatic disease; failure on at least one previous line of platinum-based chemotherapy	Nivolumab 3 mg/kg every 2 weeks on a 4-week cycle until disease progression	N/a	44	21%	59	19
Fang et al.	1	Recurrent or metastatic disease; failure on at least one previous line of platinum-based chemotherapy	Camrelizumab at escalating doses of 1 mg/kg, 3 mg/kg, and 10 mg/kg, and a bridging dose of 200 mg per dose once every 2 weeks	N/a	93	34%	NR	27
Fang et al.	1	Recurrent or metastatic disease; treatment-naive patient	Camrelizumab 200 mg (day 1), gemcitabine 1 g/m^2^ (days 1 and 8), and cisplatin 80 mg/m^2^ (day 1) every 3 weeks for six cycles, followed by camrelizumab 200 mg maintenance once every 3 weeks	N/a	23	91%	NR	61
Phase 3 preliminary data								
Xu et al.	3	Recurrent or metastatic disease; treatment-naive patient	JS001 (Toripalimab) combined with gemcitabine and cisplatin given every 3 weeks in 3-week cycles	Placebo combined with gemcitabine and cisplatin given every 3 weeks in 3-week cycles	289	NR	In progress	49% vs. 28%
Phase 3 ongoing trials								
						Estimated completion		
Merck Sharp & Dohme Corp.	3	Recurrent or metastatic disease; failure on at least one previous line of platinum-based chemotherapy	Pembrolizumab 200 mg every 3 weeks on a 3-week cycle until disease progression or unacceptable toxicity or a maximum of up to 35 cycles	Capecitabine 2000 mg/m^2^ d1-14 of each 3-week cycle or gemcitabine 1250 mg/m^2^ d1, d8; of each 3-week cycle or docetaxel 75 mg/m^2^ d1 of each 3-week cycle until disease progression or unacceptable toxicity	233	28 May 2022		
Zhang et al.	3	Recurrent or metastatic disease; treatment-naive patient	Camrelizumab 200 mg (day 1), gemcitabine 1 g/m^2^ (days 1 and 8), and cisplatin 80 mg/m^2^ (day 1) every 3 weeks for six cycles	Placebo combined with gemcitabine 1 g/m^2^ (days 1 and 8), and cisplatin 80 mg/m^2^ (day 1) every 3 weeks for six cycles	250	1 Nov 2021		
Ma et al.	3	Stage III-IVA disease (except T3-4N0 and T3N1); completed induction chemotherapy of gemcitabine and cisplatin followed by concurrent cisplatin-radiotherapy; 4–6 weeks after chemoradiation	Adjuvant: camrelizumab 3 mg/kg (≤200 mg) d1; q4wks × 12	Observation	442	1 Feb 2024		
Ma et al.	3	Stage III-IVA disease (except T3–4N0 and T3N1)	Induction: gemcitabine 1000 mg/m^2^ d1, d8; cisplatin 80 mg/m^2^ d1; sintilimab 200 mg d1; q3wks × 3; concurrent: cisplatin 100 mg/m^2^ d1; q3wks × 2; sintilimab 200 mg d1;q3wks × 3	Induction: gemcitabine 1000 mg/m^2^ d1, d8; cisplatin 80 mg/m^2^ d1; q3wks × 3; Concurrent: cisplatin 100 mg/m^2^ d1; q3wks × 2	417	1 Jan 2025		
